# Maternal Separation Enhances Conditioned Fear and Decreases the mRNA Levels of the Neurotensin Receptor 1 Gene with Hypermethylation of This Gene in the Rat Amygdala

**DOI:** 10.1371/journal.pone.0097421

**Published:** 2014-05-15

**Authors:** Hiroyuki Toda, Shuken Boku, Shin Nakagawa, Takeshi Inoue, Akiko Kato, Naoki Takamura, Ning Song, Masashi Nibuya, Tsukasa Koyama, Ichiro Kusumi

**Affiliations:** 1 Department of Psychiatry, National Defense Medical College, Tokorozawa, Japan; 2 Department of Psychiatry, Hokkaido University Graduate School of Medicine, Sapporo, Japan; 3 Department of Psychiatry and Behavioral Sciences, Albert Einstein College of Medicine, Bronx, New York, United States of America; 4 Pharmaceutical Laboratories, Dainippon Sumitomo Pharma Co. Ltd., Osaka, Japan; 5 Department of Psychiatry, The first hospital of China Medical University, Shenyang, China; Radboud University, Netherlands

## Abstract

Stress during postnatal development is associated with an increased risk for depression, anxiety disorders, and substance abuse later in life, almost as if mental illness is able to be programed by early life stressors. Recent studies suggest that such “programmed” effects can be caused by epigenetic regulation. With respect to conditioned fear, previous studies have indicated that early life stress influences its development in adulthood, whereas no potential role of epigenetic regulation has been reported. Neurotensin (NTS) is an endogenous neuropeptide that has receptors densely located in the amygdala and hippocampus. Recently, NTS systems have constituted an emerging target for the treatment of anxiety. The aim of the present work is to clarify whether the NTS system is involved in the disturbance of conditioned fear in rats stressed by maternal separation (MS). The results showed that MS enhanced freezing behaviors in fear-conditioned stress and reduced the gene expression of NTS receptor (NTSR) 1 but not of NTS or NTSR2 in the amygdalas of adult rats. The microinjection of a NTSR1 antagonist into the amygdala increased the percentage of freezing in conditioned fear, whereas the microinjection of NTSR1 agonist decreased freezing. These results suggest that NTSR1 in the amygdala may play a role in the effects of MS on conditioned fear stress in adult rats. Moreover, MS increased DNA methylation in the promoter region of NTSR1 in the amygdala. Taken together, MS may leave epigenetic marks in the NTSR1 gene in the amygdala, which may enhance conditioned fear in adulthood. The MS-induced alternations of DNA methylation in the promoter region of NTSR1 in the amygdala may be associated with vulnerability to the development of anxiety disorders and depression in adulthood.

## Introduction

Past clinical studies have shown that exposure to stress during the postnatal development periods is associated with an increased risk for depression, anxiety disorders, and substance abuse later in life [1], [2]. Similar to humans, other mammals suffering from early life stress (ELS) in the postnatal period have a vulnerability towards anxiety states and depression-like syndromes [3]–[7]. These findings suggest that variations in one's early environment may be associated with changes in gene expression and biological function that persist into adulthood. Such “programmed” effects may derive from epigenetic regulation, which causes structural alterations in genomic DNA [4], [8].

The conditioned fear stress (CFS) paradigm is based on Pavlovian aversive conditioning. An emotionally neutral stimulus (e.g., a tone, figure, light, or context) is paired with an emotionally potent and innately aversive unconditioned stimulus (e.g., an electric shock) during a conditioning phase. The assessment of conditioning then involves measuring a conditioned response elicited by the neutral stimulus. CFS is regarded as a psychological stress without physical stimuli and as a simple animal model of anxiety or fear [9]–[12]. With respect to conditioned fear, previous studies have indicated that ELS continues its influence into adulthood [13], [14]; however, no potential role of epigenetic regulation in fear learning and memory caused by ELS has been reported.

Our previous study using a DNA microarray showed that neurotensin (NTS) is the only gene that had its expression changed by CFS. This change in NTS can be overcome by a selective serotonin reuptake inhibitor treatment, which is also effective for the treatment of various anxiety disorders [15]. NTS is an endogenous neuropeptide that closely interacts with monoamine neurotransmitter systems [16], and NTS receptor (NTSR) 1 and 2 are densely located in structures that are important for anxiety, including the amygdala (AMY) and hippocampus (HIP) [17]. The systemic administration of a NTSR1 agonist significantly decreased conditioned footshock-induced ultrasonic vocalization in rats [18], even while decreasing fear-potentiated startling in rats [19]. Moreover, NTSR1 knockout mice showed higher freezing rates than wild-type mice in contextual fear stress situations [11]. However, a role for NTS in conditioned fear induced by ELS has not been documented.

The aim of the present work is to clarify whether the NTS system is involved in the disturbance of conditioned fear in rats stressed by maternal separation (MS), which is one of the most commonly used procedures for inducing ELS in rodents. Our results showed that the MS and NTSR1 antagonist enhanced freezing behaviors in conditioned fear stress. In addition, MS reduced NTSR1 gene expression and increased DNA methylation in the promoter region of AMY.

## Materials and Methods

### Animals

Adult male Sprague–Dawley (SD) rats, each weighing 230–270 g, were obtained from the Shizuoka Laboratory Animal Center (Shizuoka, Japan) and were housed in groups of four. Timed pregnant SD rats (Shizuoka Laboratory Animal Center) were delivered on gestation day 14 and were single-housed. All rats were housed in standard animal cages and received ad libitum access to food and water in a temperature-controlled environment (22±1°C) on a 12 h light/dark cycle (light phase: 0600–1800). All procedures were approved by the Hokkaido University Graduate School of Medicine Animal Care and Use Committee and complied with the Guide for the Care and Use of Laboratory Animals, Hokkaido University Graduate School of Medicine.

### MS procedure

MS was conducted according to the method of Plotsky and Meaney [20]. When a litter was born between 0900 and 1700, its day of birth was designated as postnatal day (PND) 0. When a litter was born between 1700 and 0900, its day of birth was designated as PND 1. After the pups were cross-fostered to minimize litter differences, eight males and two females were selected on PND 2. Each litter was assigned for either MS or the animal facility rearing (AFR) groups. Handling and separation were performed from 0930–1230 daily from PND 2 to PND 14. In the MS group, a dam was removed from the cage and placed into an individual cage during the separation period. The pups were then removed from the cage and placed into a clean plastic cage lined with wood-chip bedding. Then, they were taken to another room and placed in an incubator set to maintain an ambient temperature at 27– 30°C for 3 h. At the end of the separation period, the pups were returned to the cage, and then, the dam was returned. Pups in the MS group were permitted to huddle with their littermates during the separation period. AFR rats were briefly handled once a week by an animal care technician. Only male pups were used for the present study. Behavioral, molecular, and neurochemical experiments were performed during the postadolescent period (10–14 weeks old).

### Dexamethasone/corticotropin releasing hormone test

The dexamethasone (DEX)/corticotropin releasing hormone (CRH) test was conducted according to the method described by Hatzinger et al. [21]. Surgery was performed under sodium pentobarbital (40 mg/kg, i.p.) anesthesia using aseptic procedures. Rats were chronically catheterized in the jugular vein for subsequent blood sampling six days before the experiment. The catheter was exteriorized at the neck of the animal and filled with sterile saline containing gentamicin (30,000 IU/rat); 0.2 ml was infused into the animal. Rats were weighed at 0700 on the day of the experiment. The jugular venous catheter was connected via free-moving devices (Eicom, Kyoto, Japan) to a plastic syringe filled with sterile heparinized saline (50 IU/ml) at 0800. DEX (Sigma, St. Louis, MO) (30 µg/kg, 0.5 ml/kg) was administered intravenously (i.v.) at 1200. Numerous 0.2-ml blood samples were collected at 1800, 1830, 1900 and 1930 to monitor the effects of the DEX treatment on the basal plasma concentrations of corticosterone during the diurnal acrophase. At 1931, human-CRH (kindly donated from Yoshitomi Pharma, Osaka, Japan) (50 ng/kg, 0.5 ml/kg i.v.) was injected. To assess the CRH-stimulated corticosterone secretion, further blood samples were taken at 1940, 2000, 2020, and 2040. Corticosterone was measured by an enzyme immunoassay using a corticosterone EIA kit (Cayman, Ann Arbor, MI).

### Open field test

The open field box (90-cm length × 90-cm width × 40-cm height) was divided into 81 squares of equal size by stripes of black paint, and the lighting at the center of the area was set to 200 lx. The arena was divided into two sections, and the outer edge of the 20-cm width was measured from the walls (outer zone) and the center square (inner zone). Rats were placed singly in the center of the field, and their behavior was recorded for 30 min. The total distance travelled and time spent in the inner zone were assessed using tracking software (LimeLight; Actimetrics, Wilmette, IL). Total movement in the field reflected their general activity, and relative movement in the inner zone was correlated with the anxious state of the rat. All tests were performed between 1400 and 1600 to minimize Circadian influences.

### Elevated plus maze

The apparatus consisted of two open arms (50 × 10 cm) and two closed arms (50 × 10 cm) surrounded by 40 cm high side walls that extended from the central platform (10 × 10 cm). The maze was elevated 50 cm above the floor, and the lighting at the center of the area was set to 200 lx. Rats were placed singly on the central platform facing an open arm, and their behavior was recorded for 5 min. The total distance moved in the maze and the number of entries for each arm were assessed using LimeLight (Actimetrics). The total distance moved in the maze was measured as the locomotor activity. The number of entries in the open arms was measured as memory-independent fear because rats innately avoid open spaces [22]. All tests were performed between 1400 and 1600 to minimize Circadian influences.

### Footshock sensitivity test

The effects of MS on footshock-induced pain were examined. Four behaviors, including vocalization, limb withdrawal of the forepaw, limb withdrawal of the hindpaw and jumping, were used as indicators of nociception. Generally, each was used as an endpoint in the hot plate procedure [23]. The rats were individually placed in a shock chamber with a grid floor (19 × 22 × 20 cm). After a 5-min adaptation period, the rats were subjected to 15 series of scrambled electric footshocks. Each series had 10-s and 40-s intervals and ranged from 0.4 mA to 3.2 mA in 0.2 mA steps, which are presented in ascending order. The responses of the rats to each shock were recorded, and the minimal intensities of the electric footshocks at which each of the four behaviors appeared were determined.

### Condition fear stress paradigm

Rats individually underwent inescapable electric footshocks for a total of 5 min in a shock chamber with a grid floor (30× 24× 30 cm, Med Associates Inc., USA) [24]. Five footshocks (2.0 mA scrambled shock, each of 30 s duration) were delivered at shock intervals of 30 s using an ENV-410 shock generator (Med Associates Inc., USA). Twenty-four hours later, the rats were exposed to the chamber without footshocks to assess their contextual fear memory for 5 min (first exposure), as measured by their freezing behaviors. On the next day, rats were exposed to the chamber for 5 min again (second exposure). During the observation period, the duration of the freezing behavior was recorded, as previously described [24]. Freezing was defined as the absence of all observable movements of the skeleton and the vibrissae, except for movements related to respiration. Rats were classified as either freezing or active according to their behavior during a 5-s period. The percentage score of freezing represents the number of 5-s periods during which the rats froze for 5 s.

### Quantitative RT-PCR Analysis

Rats were deeply anesthetized with sodium pentobarbital and decapitated. The brains were removed and washed with ice-cold phosphate-buffered saline (PBS; pH 7.4). Coronal sections with a thickness of 1 mm were cut using a Brain Slicer (Muromachi, Tokyo, Japan) and immersed into dishes containing ice-cold PBS. The regions containing AMY and HIP were dissected carefully with a blade. Total RNA was extracted with the RNeasy Lipid Tissue Mini Kit (Qiagen, Hilden, Germany) from these regions. Total RNA was converted to cDNA with the Quantitect Reverse Transcription kit (Qiagen). Quantitative PCR was performed with the SYBR GreenER qPCR SuperMix for ABI PRISM (Invitrogen) in the ABI PRISM 7600 Sequence Detection System (Applied Biosystems, Foster, CA). All standards and unknown samples were assayed in triplicate. The conditions of PCR were the following: 50°C for 2 min and 95°C for 10 min, followed by 40 cycles of 95°C for 15 s and 60°C for 1 min. The sequences of forward and reverse primers used were as follows: AGC TGG TCA TCA ATG GGA AA and ATT TGA TGT TAG CGG GAT CG for glyceraldehyde 3-phosphate dehydrogenase (GAPDH), GCT GAC CGT ATT CCA ACT CC and CAT TGC CAT GAT CGA GGA TA for NTS, AAG CAG GCA CCC TTC ATC T and GGA GGC TGG ATG GTT CTG T for NTSR, CCT GGT GAG ACA CAA GGA TG and ACG ATG GCT CTG AGA AAA CCT G for NTSR2. GAPDH was used as a control. PCR reaction assays for unknown samples were performed simultaneously with the same standard samples (cDNA derived from rat HIP) to construct a standard curve. The relative concentrations of GAPDH and of NTS, NTSR1 or NTSR2 in unknown samples were calculated from this standard curve, and we calculated the ratio of the relative concentrations of NTS, NTSR1 or NTSR2 (already normalized by GAPDH expression) to the relative concentration of GAPDH.

### Analysis of DNA methylation in the promoter of NTSR1

Genome DNA of MS and AFR rats were isolated from AMY with a DNeasy Blood & Tissue Kit (Qiagen). A MethylCollector™ Ultra Kit (Active Motif, Carlsbad, CA) was used to enrich CpG methylated DNA fragments. The method was based on the Methylated CpG Island Recovery Assay (MIRA), which utilizes the high affinity of the MBD2b/MBD3L1 complex for methylated DNA. Genomic DNA was digested with Bfa I (New England Biolabs, Ipswich, MA) because the promoter region of NTSR1 contains the recognition site of Bfa I. The MBD2b/MBD3L1 protein-DNA complex was added to the DNA fragments, specifically binding to CpG-methylated DNA. These protein-DNA complexes were then captured with nickel-coated magnetic beads, and subsequent wash steps were performed to remove fragments with little or no methylation (unbound fragment; UF). The methylated DNA was then eluted from the beads in the presence of Proteinase K (eluted fragment; EF). Both UF and EF were purified using a MinElute PCR purification kit (Qiagen). PCR primers were designed to target the three DNA fragments (fragment A, B and C) within the CpG islands, which contain the promoter region of NTSR1 gene. The fragments were located −969 to −674 (fragment A), −673 to −283 (fragment B) and −273 to +263 (fragment C) from the first translational ATG codon. Quantitative PCR was performed with the SYBR GreenER qPCR SuperMix in the ABI PRISM 7600 Sequence Detection System. All samples were assayed in triplicate. The percentage enrichment, which represented the degree of methylation of each fragment, was calculated as follows: enrichment (%)  =  2^CT (UF) – CT (EF)^/(1 + 2^CT (UF) – CT (EF)^) × 100. The conditions for the PCR were the following: 50°C for 2 min and 95°C for 10 min, followed by 40 cycles of 95°C for 15 s and 60°C for 1 min. The primers used were as follows: CCG AGC CAG CTG TAC AAA G and GGC AGC ACA ATC TTC TCC TT for NTSR1-fragment A, CAA GCA GAA GAG GGA GAA CG and TGC TAC GGA CCT CCA GAT TC for NTSR1- fragment B, AAG AAG AGT GGA TCC CTG AGC and TAT GCT GCT TTG TCC TGC AC for NTSR1- fragment C.

### Microinjections

Surgery was performed using sodium pentobarbital anesthesia (40 mg/kg, intraperitoneally) under aseptic conditions. The head position was adjusted to place the bregma and lambda on the same horizontal plane in a stereotaxic frame. Naïve rats were implanted with a bilateral 26-gauge guide cannula (Plastics One, Roanoke, VA) directed toward AMY (immediately the central nucleus of AMY). The coordinates were −2.5 mm posterior to the bregma, 4.7 mm bilateral to the midline, and 7.2 mm ventral from the surface of the skull. Dummy stylets were inserted into guided cannulae, and the rats were allowed a 10–12-day post-recovery period. After surgery, the rats were housed individually. During the recovery period, the rats were handled 4 times before the microinjection procedure. PD149163, a specific agonist of NTSR1, was kindly donated by the NIMH Chemical Synthesis and Drug Supply Program (Washington D.C.) and SRI International (Menlo Park, California), and SR48692 was purchased from Tocris Biosciens (Bristol, UK). PD149163 was prepared in 0.9% physiological saline as a 1 mM stock solution, and SR48692 was prepared in DMSO as 1 mM stock solution. They were stored at −20°C. Twenty-four hours, after fear conditioning, rats were injected bilaterally into AMY with 0.5 µl/side of saline or DMSO, PD149163 (10, 100, or 1000 µM) or SR48692 (10, 100, or 1000 nM). After the removal of the dummy stylets, bilateral infusions were given simultaneously for 30 sec using 33-gauge stainless steel cannulae projecting 1.0 mm beyond the tips of the guide cannulae. Each injector was connected by polyethylene tubing to a 100-μl syringe that was driven at a rate of 1 µl/min by an infusion pump. Injectors remained in place for an additional 1 min to confine the drug to the target site. Ten minutes after the infusion, the rats were again placed in the shock chamber and observed for 5 min without shocks to assess their contextual fear memory (first exposure). On the next day, the rats were exposed to the chamber for 5 min again (second exposure). The 0.5-μl injection volume was based on a previous study [25]. The injection was aimed at the central nucleus because this area contains many NTS-immunoreactive neurons [26]. However, the entire region of AMY might be influenced by these drugs, as it has been reported that a volume of 0.5 µl is likely to infiltrate the other areas of AMY [27]. Injection placements were verified immediately after the second exposure session, as described previously [25]. Only the data from animals with the correct placement of the cannulae were analyzed.

### Statistics

Data were analyzed using SPSS Statistics version 21 (SPSS, Chicago, IL). Numerical data were analyzed by an unpaired *t*-test, two-way repeated ANOVA or one-way ANOVA, followed by Tukey's post hoc test. In all cases, the significance level was set at *p*<0.05. All data are shown as the means ± S.E.M.

## Results

### MS affected the hypothalamic-pituitary-adrenocortical (HPA) system but not body weight or anxiety behaviors

**Figure 1:**
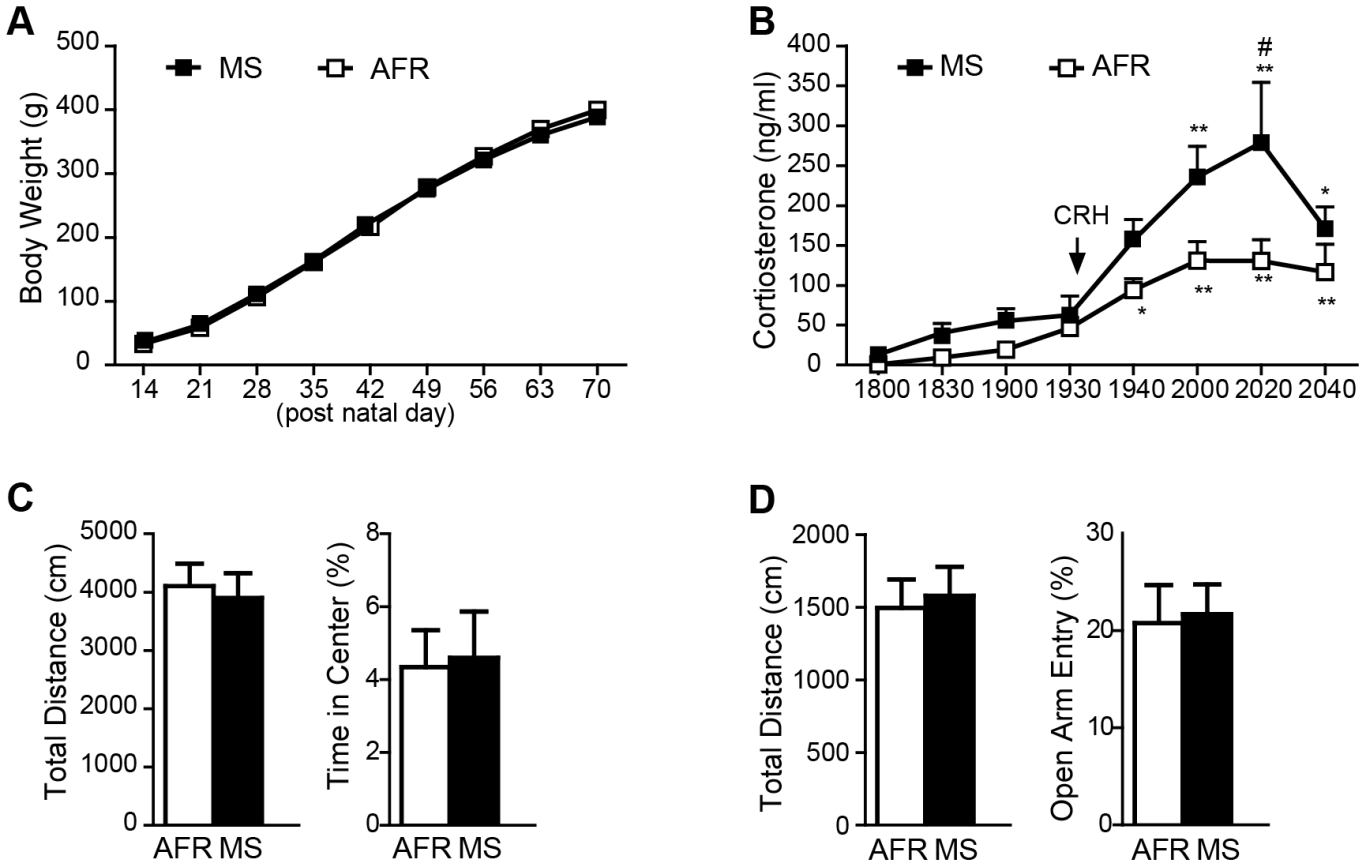
Effect of maternal separation (MS) on body weight, hypothalamic-pituitary-adrenocortical system and anxiety behaviors. *A*, Body weight change of rats in MS and animal facility rearing (AFR) groups (MS, n = 22; AFR, n = 17). *B*, Plasma corticosterone concentrations of MS (n = 8) and AFR (n = 6) rats between 1800 and 2040 h on the dexamethasone/corticotropin-releasing hormone (DEX/CRH) test. All rats were pretreated with DEX (30 µg/kg i.v.) at 1200 h and administered with CRH (50 ng/kg i.v.) at 1931 h. MS rats showed significant increases in plasma corticosterone levels (#*p*<0.05 vs AFR rats, *p<0.05, **p<0.01 vs baseline). *C*, Open field test. Total distance and % time in center during the 30 min test were analyzed. *D*, Elevated plus maze test. Total distance and % number of open arm entries during the 5 min were analyzed. Error bars represent SEM.

No significant difference was found between the AFR and MS groups in body weight at 14 PND, at which point the maternal separation procedure had finished (Fig. 1A). A two-way repeated measure ANOVA revealed that MS had no significant main effect of the group (F_(1, 62.5)_ = 0.010, *p*>0.05, Fig. 1A) and no interaction (F_(8, 447)_  = 1.758, *p*>0.05, Fig. 1A). The effect of MS on plasma corticosterone levels was examined in the DEX/CRH test (Fig. 1B). A two-way repeated measure ANOVA revealed a significant main effect of group (F_(1, 84)_  = 5.389, *p*<0.05) and time (F_(7, 84)_  = 15.75, *p*<0.001) but no significant interaction between group and time. The release of corticosterone stimulated by CRH was significantly greater in MS rats than in AFR rats, as was reflected by the higher AUC values (MS: 14650±2584; AFR: 8032±1372; *p*<0.05). Tukey's post hoc test revealed that the maximum rise of corticosterone was significantly greater in MS over AFR rats (*p*<0.05). Basal corticosterone levels between 1800 and 1930 h did not differ between the MS and AFR rats.

We examined the effects of MS on memory-independent anxiety behaviors in the open field test and elevated plus maze test. In the open field test (Fig. 1C), no significant difference was found between the two groups in the total distance moved in the field or in the % time spent in the center field. In the elevated plus maze (EPM) test (Fig. 1D), no significant difference was found between the two groups in the total distance moved in the maze or in the % of entries into the open arms.

### MS enhanced freezing behavior in CFS

**Figure 2:**
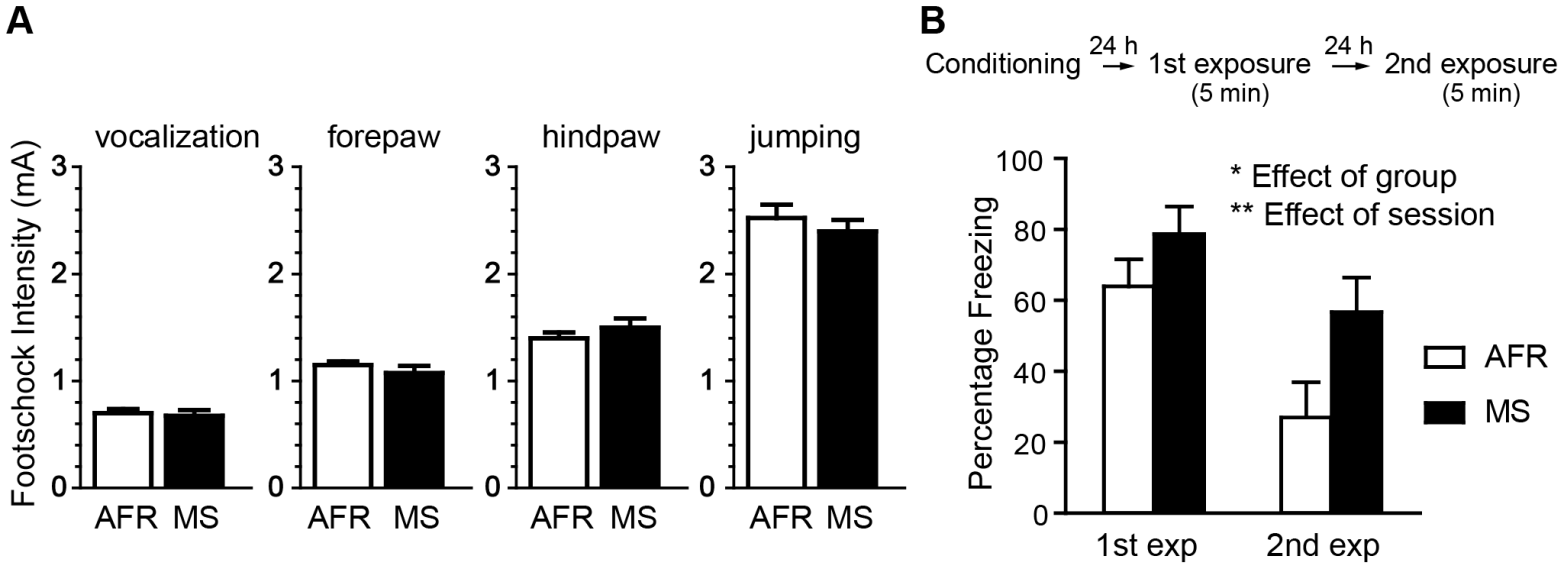
Effect of MS on freezing behavior in conditioned fear stress (CFS). ***A***, Sensitivity to pain induced by electrical stimulation. The minimal levels of the current required to elicit the stereotypical response of vocalization, limb withdrawal of the forepaw, limb withdrawal of the hindpaw and jumping were determined. Data are represented as pain thresholds (mA) (n = 8 per each group). ***B***, Freezing rates of the MS and the AFR rats in the first exposure of 24 h after and the second exposure of 48 h after the fear conditioning by a 2.0 mA footshock. Two-way repeated ANOVA revealed significant main effects of the group (*p<0.05) and session (**p<0.01) but no significant interactions between the group and session (n = 10–12 per group).

Figure 2A shows the minimal intensities of the electric footshocks at which pain-related behaviors first appeared. There were no significant differences between the two groups in vocalization, limb withdrawal of the forepaw, limb withdrawal of the hindpaw or jumping. These data indicate that the sensitivity to the footshock was not different between the AFR and MS groups. Next, we examined memory-dependent behaviors in CFS (Fig. 2B). Twenty-four hours after the conditioning session for the 2.0 mA footshock, a two-way repeated ANOVA revealed a significant main effect for the group (F_(1, 19)_  = 5.25, *p*<0.05) and session (F_(1, 19)_  = 37.17, *p*<0.01) and no significant interaction between the group and session.

### MS decreased the mRNA expression of NTSR1 in AMY

**Figure 3:**
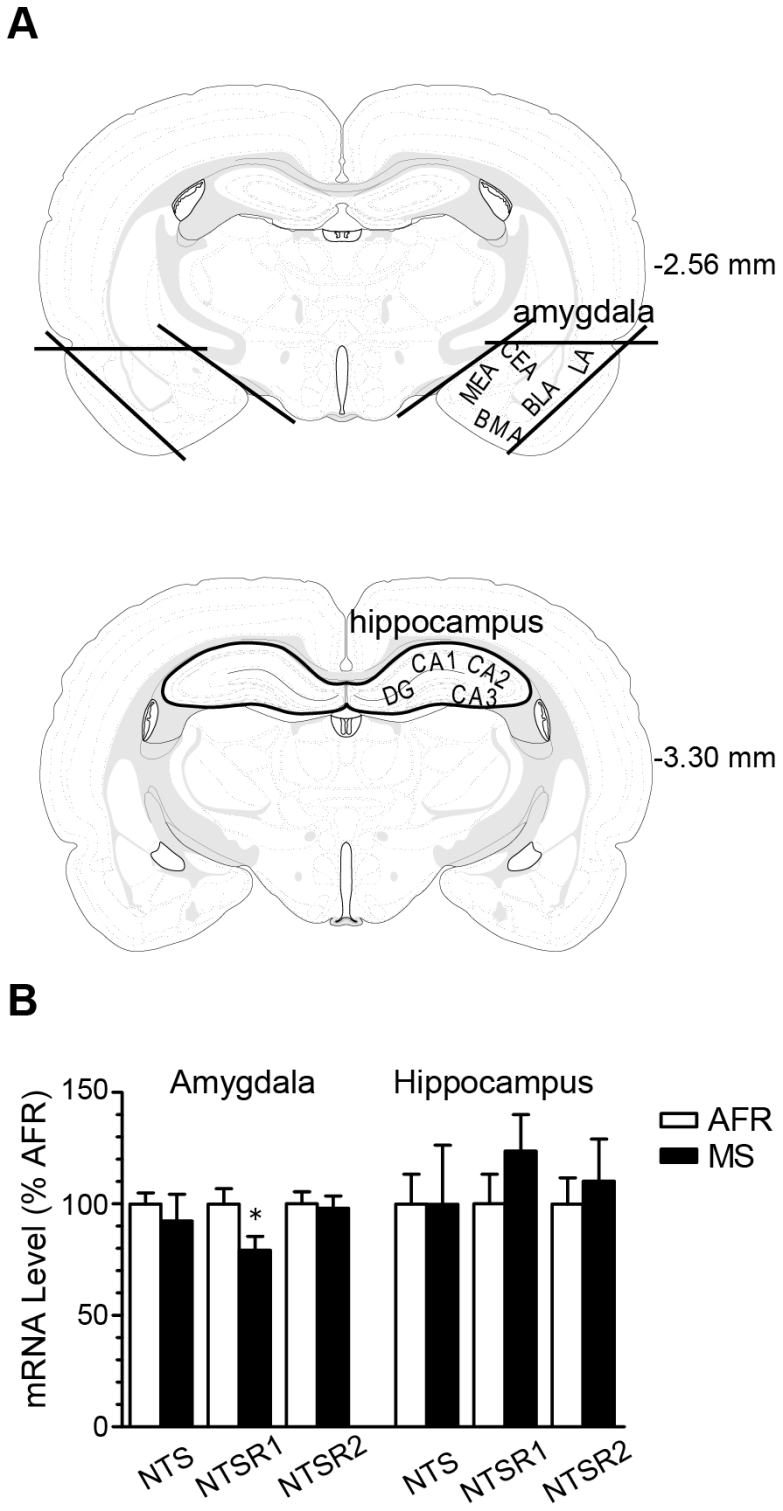
Effect of MS on the mRNA expression of neurotensin receptor 1 (NTSR1). ***A***, Dissected regions of the amygdala (AMY) and hippocampus (HIP) (heavy black line) in coronal sections with a thickness of 1 mm from the rat brain atlas of Paxinos and Watson [69] (2.56 and 3.30 mm posterior to bregma). ***B***, Comparative mRNA expression levels of neurotensin, NTSR1 and NTSR2 in the AMY and HIP of MS and AFR rats. NTSR1 mRNA levels in MS rats were significantly decreased in AMY compared to AFR rats (n = 7 – 9 per group; *p<0.05, **p<0.01). Error bars represent SEM.

AMY and HIP play a central role in fear learning and memory [25], [28]. Previous findings have suggested that the NTS system is involved in fear memory processes [18], [29], [30]. Therefore, we examined the effects of MS on the mRNA expressions of NTS, NTSR1 and NTSR2 in AMY and HIP with quantitative RT-PCR. Figure 3A indicates the dissecting regions as HIP (upper coronal section) and AMY (lower coronal section). The bilateral AMY and HIP of adult rats were dissected, as indicated with heavy black lines. Figure 3B shows the mRNA expression levels of NTS, NTSR1 and NTSR2 in AMY and HIP of MS or AFR rats. In AMY, a significant decrease in mRNA expression of NTSR1 was observed in MS rats (*p*<0.05), but no significant differences were found between the two groups in the mRNA expressions of NTS or NTSR2. In HIP, no significant differences were found between the two groups in mRNA expressions.

### Microinjections of NTSR1 antagonist or agonist in AMY showed opposite effects on freezing behaviors in CFS

**Figure 4:**
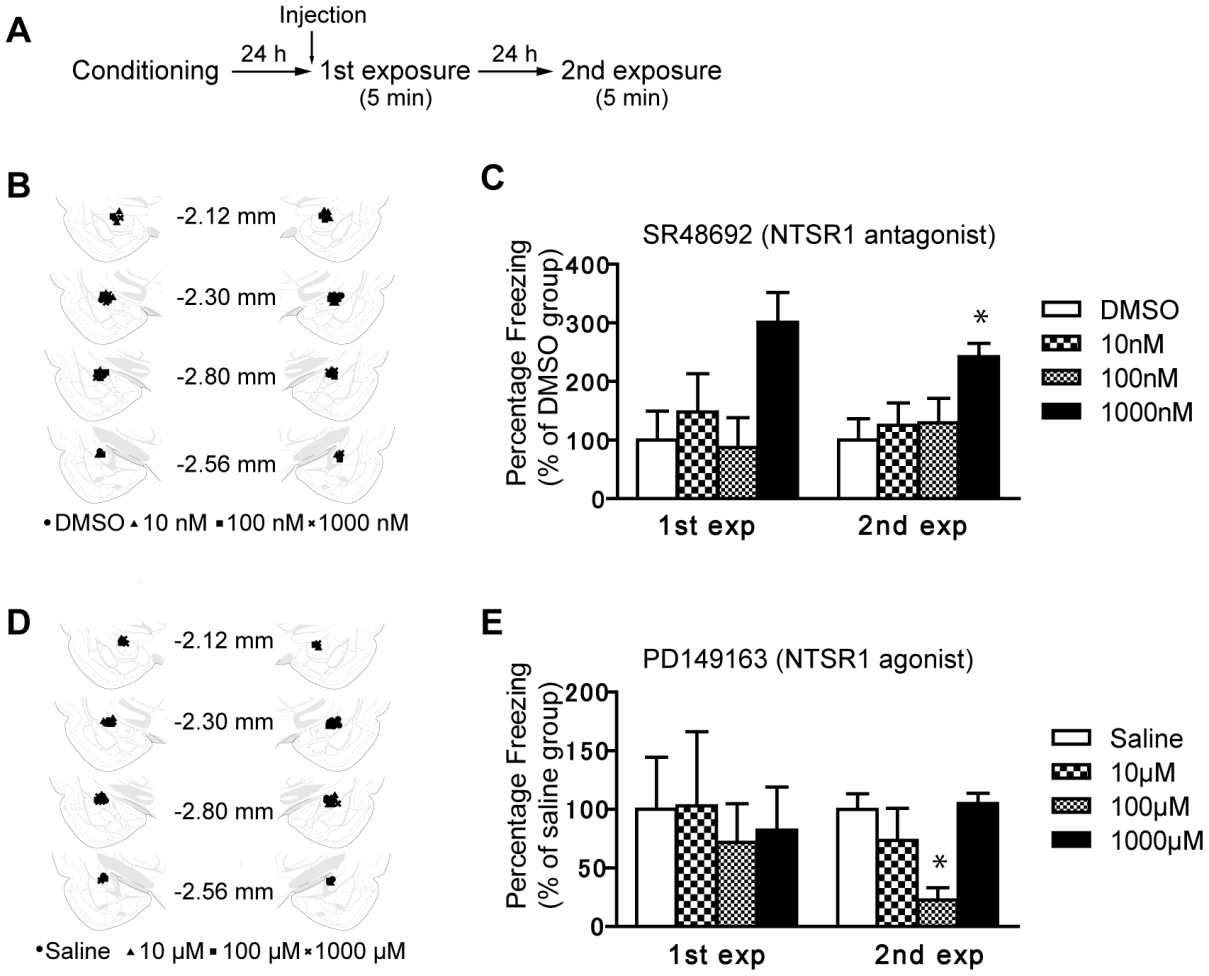
Effect of the NTSR1 agonist and antagonist in AMY on CFS. ***A***, Experimental design. ***B***, ***D***, Location of the needle tips for intra-AMY infusions of SR48692 (B) and PD149163 (D). Brain structure diagrams of coronal sections through AMY are adapted from the rat brain atlas of Paxinos and Watson [69] (2.12 – 2.56 mm posterior to bregma). ***C***, Bilateral infusion of an NTSR1 antagonist (SR48692) dissolved in 0.5µl of DMOSO in AMY produced a significant increase in the freezing rate at a concentration of 1000 nM in the second exposure. ***E***, Bilateral infusions of a NTSR1 agonist (PD149613) dissolved in 0.5 µl of saline into AMY produced a significant decrease in the freezing rate at a concentration of 100 µM at the second exposure (n = 7 – 8 rats per all groups; *p<0.05 vs. saline or DMSO control). Error bars represent SEM.

To determine whether NTSR1 in AMY is critically involved in CFS, we examined the effects of microinjections of an antagonist or agonist of NTSR1 into AMY of naïve rats on CFS. SR48692, which has a higher affinity for NTSR1 compared to NTSR2 [31], was used as an antagonist of NTSR1. PD149163, which selectively binds to the NTSR1 with no affinity for NTSR2 [32], was used as an agonist of NTSR1. CFS and microinjections of SR48692 or PD149163 were applied, as shown in Fig. 4A. The cannula placements for rats injected with SR48692 and PD1491633 into AMY are shown in Figures 4B and 4D, respectively. Tissue damage was not apparent in either of the treatment groups or in the saline groups.

Figure 4C shows the freezing rate (% the DMSO group) of rats with the bilateral injections of saline or SR48692 (10 nM – 1000 nM) into AMY. One-way ANOVA indicated the significant effects of drug treatment at the first exposure (F_(3, 27)_  = 3.26, *p*<0.05) and at the second exposure (F_(3, 27)_  = 3.19, *p*<0.05). Tukey's post hoc test showed that all drug treatment groups were not significantly higher than the DMSO group at the first exposure (*p*<0.05), but the freezing rate at a dose of 1000 nM was significantly higher than that of the DMSO group at the second exposure (*p*<0.05). Figure 4E shows the freezing rate (% the saline group) of the rats with bilateral microinjections of saline or PD149163 (10 µM – 1000 µM) into AMY. One-way ANOVA indicated no significant effect of drug treatment in the first exposure (F_(3, 25)_  = 0.104, *p*>0.05) but a significant effect in the second exposure (F_(3, 25)_  = 5.45, *p*<0.05). Tukey's post hoc test showed that the freezing rate at a dose of 100 µM was significantly lower than that with saline at the second exposure (*p*<0.05). These data indicate that the microinjection of a NTSR1 agonist (PD149163) into AMY disrupted the conditioned freezing and that the microinjection of NTSR1 antagonist (SR48692) into AMY potentiated it.

### MS increased DNA methylation in the promoter region of the NTSR1 gene in AMY

MS decreased the expression of NTSR1 mRNA in adulthood (Fig. 3B). Such long-lasting effects are often mediated by epigenetic mechanisms, including DNA methylation [33]. Therefore, we examined whether MS affected DNA methylation in the promoter region of the NTSR1 gene in AMY.

**Figure 5:**
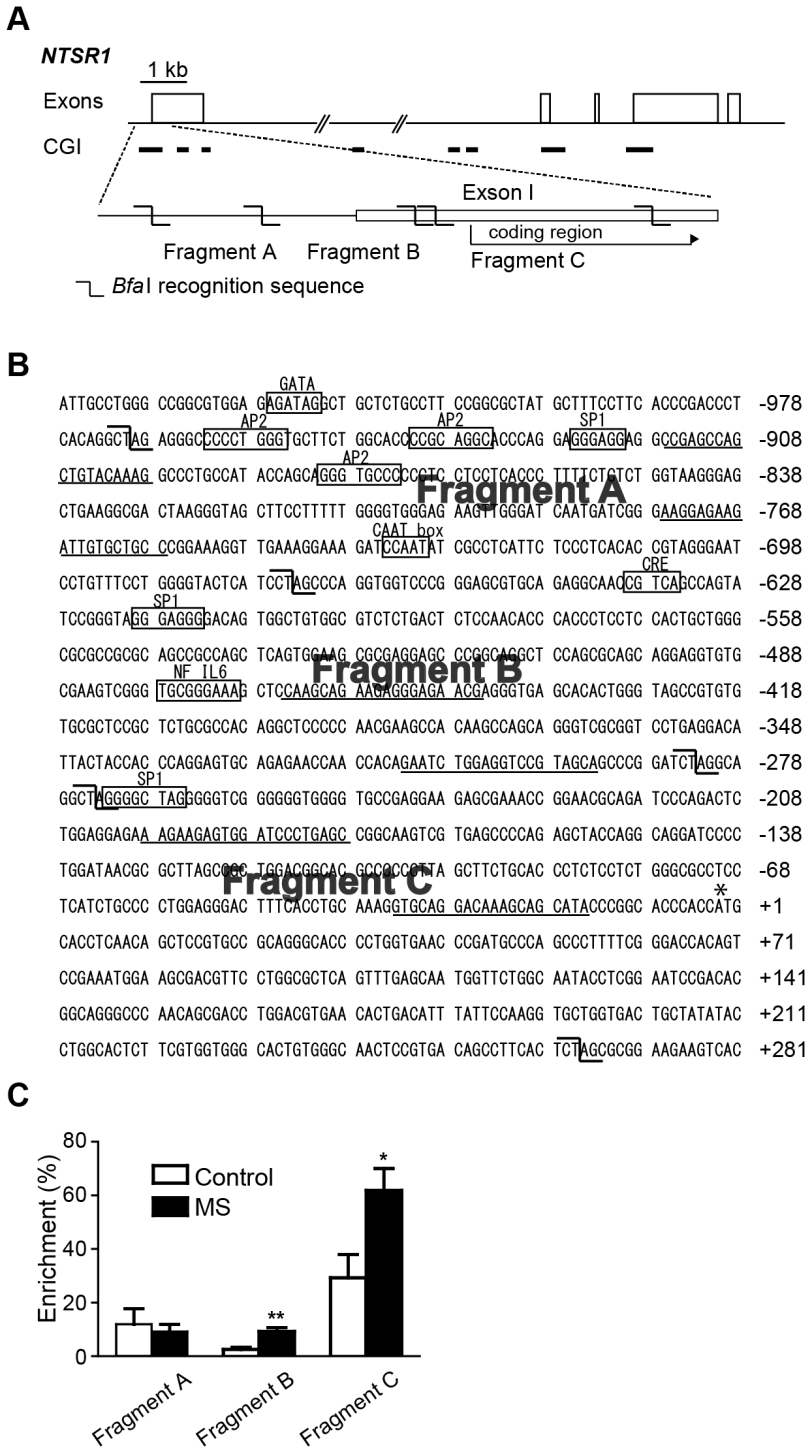
Effect of MS on DNA methylation in the NTSR1 promoter region in AMY. ***A***, The diagram of NTSR1 DNA and the locations of the CpG islands (CGI) and fragments A, B and C. ***B***, Nucleotide sequence of the promoter region of the rat NTSR1 gene [34]. The hooked shape indicates the Bfa I recognition sites. Underlined sequences indicate the position of the primers of fragments A, B and C. The potential binding sites for transcription factors, GATA, activating protein 2 (AP2), specificity protein 1 (SP1), CAAT box, cAMP response elements (CRE), and nuclear factor-interleukin 6 (NF IL6) are marked by boxes, and their names are indicated above. The coding sequence of the first exon is indicated by an asterisk. ***C***, Methylation analysis of NTSR1 promoter region using a MethylCollector Ultra Kit (Active Motif). The percentage enrichment was calculated as follows: enrichment (%) = 2^CT (UF) – CT (EF)^/(1 + 2^CT (UF) – CT (EF)^) × 100. The eluted fragment (EF) represents a DNA fragment with more than five methylated CpG sites, and the unbounded fragment (UF) represents a DNA fragment with four or fewer methylated CpG sites. The % enrichment of fragments B and C was significantly increased in the MS group compared with the AFR group (n = 8 per group; *p<0.05 vs. AFR, **p<0.01 vs. AFR, unpaired *t*-test).

Figure 5A illustrates a diagram of the NTSR1 gene and the sites of CpG islands. Figure 5B shows nucleotide sequences of the promoter region of NTSR1 and the potential binding sites for following transcription factors: GATA, activating protein 2 (AP2), specificity protein 1 (SP1), CAAT box, cAMP response elements (CRE), and nuclear factor-interleukin 6 (NF IL6) [34]. The coding sequence of the first exon is indicated by an asterisk. A large G+C-rich domain with characteristics in common with a CpG island (64.5% over 700 nt: −648 to 52) extended from the first translation ATG codon (+1) in the promoter region and into part of the coding region of the NTSR1 gene. The promoter region between −662 and −470 from the first translation codon contained strong positive regulatory elements that drove the expression of NTSR1 gene [34]. This region lacked a typical TATA or CAAT box but contained a CRE-like half site and putative Sp1 binding sites, suggesting the possible involvement of these factors in the positive regulation of NTSR1 expression. The locations of the fragments A, B and C are indicated in figures 5A and 5B. The underlined sequences in figure 5B show the position of the primers of fragment A, B and C. The % enrichment represents the degree of methylation of each fragment. The analysis of real time PCR revealed that the % enrichment of the fragments B and C was significantly increased in the MS group compared with the AFR group (unpaired *t*-test; *p*<0.01 and *p*<0.05, respectively) (Fig. 5C).

## Discussion

We have shown that MS enhances conditioned fear in adulthood and increases the DNA methylation in the promoter region on the NTSR1 gene in AMY. In addition, the pharmacological blockade of NTSR1 in AMY activated conditioned fear, whereas the activation of NTSR1 disrupted it. These findings suggest that MS may enhance conditioned fear via DNA methylation of the promoter region of NTSR1 in AMY, underlying its decreased mRNA expression.

Our experiment showed that a remarkable increase in corticosterone levels following CRH stimulation could be observed with the DEX/CRH test in MS group, affirming that a long-term dysregulation of the HPA axis is induced by ELS in animal and human studies [33], [35]–[37]. MS did not affect the body weight, anxiety behaviors in the elevated plus maze test or locomotor activity in the open field test. Otherwise, the freezing rate of MS rats was higher than that of AFR rats in the context of exposure without the different sensitivities to pain induced by footshock. Taken together, it has been suggested that MS may disturbance the processes of conditioned fear memory in adulthood. Memory retrieval may initiate two potentially dissociable but opposite processes: reconsolidation and extinction [38]–[40]. Previous studies have shown that the length of re-exposure determines, in part, which of these two is dominant. Brief re-exposure to a conditioning stimulus seems to trigger a second wave of memory consolidation (reconsolidation), whereas prolonged exposure to the conditioning stimulus leads to the formation of a new memory that competes with the original memory (extinction) [39]–[46]. However, other characteristics of a memory, such as its age and strength, also affect behaviors after memory retrieval. Hence, our results lead us to hypothesize that MS enhanced the reconsolidation or decreased the extinction of fear memory because our experiments were not sufficient to discriminate between these two processes.

Studies in developmental psychobiology and physiology are replete with examples of the environmental programming of gene expression. Such studies have commonly reported that a variation in the early environment is associated with changes in gene expression and function that persist into adulthood and thus well beyond the duration of the relevant environmental event [5], [6], [47]. Our results showed that MS decreased the expression of NTSR1 mRNA in AMY, but not in HIP, in adulthood. In addition, the pharmacological blockade of NTSR1 in AMY increased the freezing rate in the second exposure to CFS, whereas the activation of NTSR1 decreased the freezing rate. These results suggest the involvement of NTSR1 located at AMY in conditioned fear. The systemic administration of an NTSR1 agonist was reported to have an anxiolytic profile in a conditioned footshock-induced ultrasonic vocalization model [48]. NTSR1 knockout mice showed a higher freezing rate than wild-type mice in contextual fear memory [49]. Our study using microinjections of NTSR1 agonist or antagonist to the AMY agrees with these past studies.

It has been shown that NTS and its receptor play a role in pain transmission [50]–[54]. This may indicate that NTSR1 agonism or antagonism can change the freezing rate in CFS by modulating the pain transmission instead of affecting the fear memory. To exclude this possibility, NTSR1 agonists or antagonists were microinjected before the first exposure, but they did not influence the threshold of pain induced by footshock in the conditioning session. Therefore, it is unlikely that the observed effects on learning fear memory are due to changes in the pain threshold or other nonspecific performance variables associated with memory acquisition. The concentration of PD149163 (NTSR1 agonist) used in this study was in the range of pmol (10 pmol –1000 pmol), comparable to the dose range used by others for intracerebral injections [51], [52]. On the other hand, the concentration of SR48692 (NTSR1 antagonist) was in the range of fmol (10 fmol–1000 fmol). Because SR 48692 has an agonistic effect at high doses [55], [56], we used a low concentration of SR48692 compared to PD149163, which was also comparable to the dose range used by others for intracerebral injections [29], [51], [57], [58].

We examined the effect of MS on DNA methylation in the promoter region of the NTSR1 gene in AMY. DNA methylations of fragments B and C were significantly increased in the MS group. Fragment B is located 673–283 bp upstream from the first translational codon, which includes the core promoter region of the NTSR1 gene. Increased methylation of CpG dinucleotides, particularly in the 5′-promoter regions, is generally associated with a decrease in gene expression [59]. Therefore, these results suggest that DNA hypermethylation at the promoter region of the NTSR1 gene decreased mRNA expression. Whereas the function of this hypermethylation of the coding regions is not clear, it was difficult to explain why the methylation of the fragment C in MS rats was increased. Taken together, MS enhanced conditioned fear in adulthood, which may be induced from the DNA methylation of the promoter region of the NTSR1 gene in AMY.

Recent studies suggest that the DNA methylation induced by ELS plays a role in the development of psychiatric illness [36], [60]. Moreover, human studies have proven that individuals who are exposed to ELS are more likely to develop post-traumatic stress disorder (PTSD) and phobias [61]–[65]. Our results suggest the possibility that the DNA methylation of the promoter region of NTSR1 gene in AMY may induce a vulnerability to anxiety disorders, such as PTSD and phobia, as disturbances of the fear memory processes play an important role in the development of these diseases [9], [66], [67]. However, we were unable to verify whether the relationship between the MS-induced down-regulation of NTSR1 mRNA and impaired fear memory processes was causative or correlative. This is one important limitation of this study. To prove this, for example, we would have to confirm the overexpression of NTSR1 in AMY of MS rats recovering from the MS-enhanced conditioned fear memory. The other limitation of this study is that we did not examine the protein levels of NTSR1. Various genes have been reported to be associated with fear memory. For example, the decrease in the GluN2B subtype of the NMDA subunit of the glutamate receptor in AMY would serve to inhibit the reconsolidation of the contextual memory [68]. Thus, it was possible to be associated with other genes than NTSR1 in the processes of fear memory in this study. Additionally, we examined the effect of drug microinjection before the first exposure test to clarify the role of NTSR1 in fear because the effect of drugs on the expression of conditioned fear anticipates the drug's effect on this response in many cases [11]. However, we did not administer the agonist or antagonist of NTSR1 before fear conditioning. Accordingly, future studies to examine the effect of NTSR1 agonism and antagonism administered before fear conditioning or before both fear conditioning and first exposure are needed.

In conclusion, we showed that MS leaves epigenetic marks in the promoter region of the NTSR1 gene in AMY that may enhance conditioned fear memory in adulthood. These results provide the first evidence that differential DNA methylation in the promoter region of NTSR1 gene in AMY may be associated with the conditioned fear memory, although epigenetic alteration is a correlate, and a no cause-effect relation can be established by this study. Because ELS plays an important role in the clinical vulnerability to the development of anxiety disorders and depression in adulthood, our results suggest the hypothesis that manipulating the NTSR1 gene in AMY may have a therapeutic action to improve from these diseases. To clarify this hypothesis, it would be useful to investigate whether the overexpression of NTSR1 in AMY of MS rats can recover MS-enhanced conditioned fear memory.
